# Dyspnea and Wheezing: Still a Challenge for Pulmonologists

**DOI:** 10.1155/2012/610949

**Published:** 2012-12-22

**Authors:** Ana Sofia Castro, Ana Barroso, Sara Conde, Bárbara Parente

**Affiliations:** Pulmonology Department, Centro Hospitalar Vila Nova Gaia-Espinho (CHVNG-E), 4434-502 Vila Nova de Gaia, Portugal

## Abstract

Schwannoma is a neurogenic tumor originating from the nerve sheath Schwann cells. Intrathoracic location is rare, and the endobronchial location is exceptional. Schwannoma is a rare tumor; the majority of lesions are benign and usually asymptomatic. The authors present a case report of a 83-year-old woman, nonsmoker, observed in the emergency department for wheezing and cough lasting for 2 months. Chest tomography showed a right hilar pulmonary mass, ill defined, with thick and irregular walls, centered on the upper lobe bronchus, which was obliterated. Fiberoptic bronchoscopy showed a necrotic mass obstructing the right upper lobe bronchus whose biopsy allowed the diagnosis of benign schwannoma. Subsequently, the patient carried tumor ablation by laser bronchoscopy, with the resolution of the respiratory symptoms. This case stands out for its rarity but also because it is an excellent example of the importance of endoscopic techniques for therapeutic purposes. Schwannoma is a benign tumor in which surgical or endoscopic intervention generally prevents local recurrence and associated clinical manifestations.

## 1. Introduction 

Schwannoma is a neurogenic tumor originating from nerve sheath Schwann cells. Intrathoracic location is rare, involving essentially the mediastinum, being its endobronchial location exceptional. Schwannoma is a rare tumor. The majority of lesions are benign and usually asymptomatic.

## 2. Case Report

A female patient, 83 years old, nonsmoker, was observed in the emergency department for wheezing and nonproductive cough for about 2 months. Her medical history revealed hypertension for the last 10 years; family history was unremarkable.

Pulmonary auscultation showed decreased breath sounds in the upper third of the right hemithorax, without the reduction of vocal vibrations. Physical examination did not show any other relevant changes.

**Figure 1:**
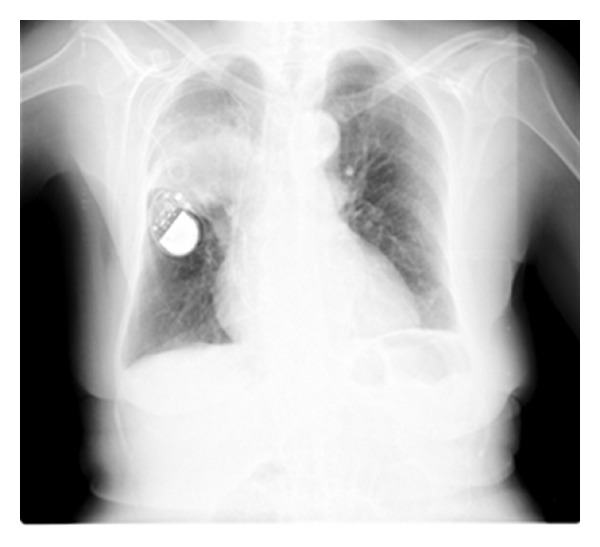
Chest X-ray revealing a heterogeneous hypotransparency in the right hemithorax upper third.

Chest X-ray revealed a heterogeneous hypotransparency in the right hemithorax, upper third; blood count and blood biochemistry unchanged (Figure 1).

**Figure 2:**
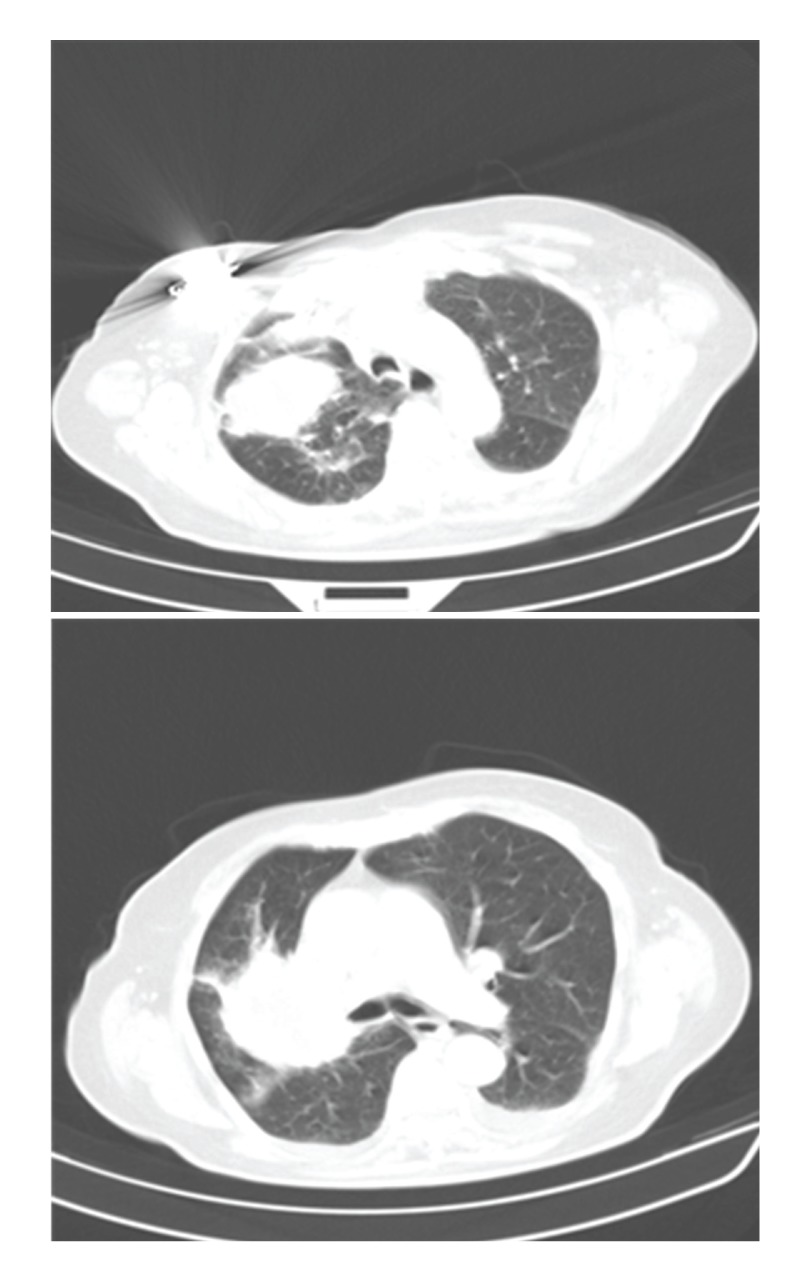
Chest tomography showing a suprahilar right lung mass, ill defined, thick walled, and irregular, centered in upper lobe bronchus, which is obliterated, probably due to tumor necrosis.

To clarify radiological findings, a chest tomography (CT) was performed that showed a suprahilar right lung mass, ill defined, thick walled, and irregular, centered in upper lobe bronchus, which is obliterated, probably due to tumor necrosis (Figure 2). Fiberoptic bronchoscopy revealed a necrotic mass obstructing the right upper lobe bronchus and the bronchial lavage was negative for malignant cells and mycobacteria, without any microbiological bacterial growth. The right upper lobe bronchus biopsy confirmed the diagnosis of benign Schwannoma.

Subsequently, the patient was considered for laser therapy for tumor ablation. Rigid bronchoscopy was performed and a flexible fiberoptic bronchoscope was inserted through the rigid broncoscope for laser tumor ablation. The tumor was partially removed with the patency of the right upper lobe. The broncoscopic procedure had no immediate complications and the patient was discharged 24 h later without any respiratory symptoms.

Unfortunately, no bronchoscopy or CT was done as a followup or to exclude recurrence because the patient had a fatal cerebrovascular stroke one month after the procedure.

## 3. Discussion

Schwannoma, also called neurinome or neurilemmoma, is a Schwann cells tumor, usually of benign histology, in the form of a well-circumscribed encapsulated mass arising from cranial or peripheral nerves [1, 2]. 

Bronchial location is extremely rare. Schwannoma represents 4% of tracheobronchial tumors and 0.2% of bronchopulmonary tumors [2]. 

Straus and Guckien first described three cases of tracheobronchial Schwannomas in 1951 [3], in a Japanese review. Kasahara et al. found 48 observations of neurilemmomas of the bronchial tree [4] and in 2005, Mizobuchi et al. [2] analyzed 22 cases of confirmed bronchial Schwannomas.

There are two major types of neurogenic tumors in the bronchial tree: Schwannomas and Neurofibrome. Schwannomas are well-encapsulated tumors formed by the Schwann cells sheath, distinguished in two varieties: the Antoni type A, with dense tissue, rich in cells and Antoni type B, more lax and poor in cells. In Neurofibromes, by contrast, lesions are diffuse, ill defined, formed from Schwann cells tumor proliferation and fibroblasts from nervous connective tissue. Schwannomas and Neurofibromes with bronchopulmonary location are generally benign, although malignant cases have been described [2, 5]. 

In Kasahara et al. review, Schwannomas were divided into two main types according to their location—central and peripheral. When the lesion is located proximal to the trachea or bronchus and it is visible in bronchoscopy, it is classified as central and may cause symptoms such as coughing and wheezing due to airway stenosis [4].

Endobronchial neurogenic tumors occur preferably in young adults between 20 and 30 years old, but two cases were described above the age of 80 years old [2, 5]. 

Clinical signs are not specific, since this is usually a benign and slow evolution tumor, remaining asymptomatic for long periods, manifesting problems such as breathlessness, wheezing, or cough secondary to bronchial obstruction, or possibly by hemoptysis.

Radiological changes depend on the tumor size, its location in the tracheobronchial tree, and the degree of obstruction that it determines. High-resolution CT scan allows an accurate detection of tumor topography, its density, changes in the bronchial structure, and ventilation, essential for future therapeutic attitude and followup [1]. 

Fiberoptic bronchoscopy is essential to determine the level of obstruction, morphological aspect, and implantation type and for obtaining lesion biopsies in view of the histological diagnosis [1]. Schwannomas macroscopically are nodules covered with normal bronchial mucosa. They can also be hypervascular and cause prolapse into the bronchial lumen [6].

The ideal treatment is to excise the tumor and restore the bronchial patency [5]. They can be removed endoscopically (forceps resection, electrocoagulation, and laser ablation) or by surgical resection—lobectomy or pneumonectomy [6]. 

In the presented case report, it was decided to perform a less invasive treatment with laser tumor ablation, given the patient advanced age and the risks of a major surgical intervention as a lobectomy were not negligible.

Prognosis, in general, is good after the lesion complete excision. Relapses are exceptional. Histopathology is the cornerstone of Schwannoma diagnosis and bronchoscopy is the procedure of the choice for both diagnosis and treatment. 

This case stands out for its rarity, but also because it is an excellent example of the endoscopic techniques important for therapeutic purposes.

Endobronchial Schwannoma should be present as a differential diagnosis in a patient with dyspnea, wheezing, and lung mass. It is a benign tumor and a surgical or endoscopic treatment usually prevent local recurrence and associated clinical manifestations.
